# Metabolic Patterns of High-Invasive and Low-Invasive Oral Squamous Cell Carcinoma Cells Using Quantitative Metabolomics and ^13^C-Glucose Tracing

**DOI:** 10.3390/biom13121806

**Published:** 2023-12-18

**Authors:** Wenrong Jiang, Ting Zhang, Hua Zhang, Tingli Han, Ping Ji, Zhanpeng Ou

**Affiliations:** 1 Chongqing Key Laboratory of Oral Diseases and Biomedical Sciences, Chongqing 401147, China; 501041@hospital.cqmu.edu.cn (W.J.); 501422@hospital.cqmu.edu.cn (T.Z.); 2 Chongqing Municipal Key Laboratory of Oral Biomedical Engineering of Higher Education, Chongqing 401147, China; 3 Stomatological Hospital of Chongqing Medical University, Chongqing 401147, China; 4 Ministry of Education of China International Collaborative Joint Laboratory of Reproduction and Development, Chongqing Medical University, Chongqing 400016, China; zh2844@gmail.com (H.Z.); tinglihan@cqmu.edu.cn (T.H.); 5 State Key Laboratory of Maternal and Fetal Medicine of Chongqing Municipality, Chongqing Medical University, Chongqing 400016, China; 6 Institute of Life Sciences, Chongqing Medical University, Chongqing 400016, China; 7 Department of Obstetrics and Gynecology, The First Affiliated Hospital of Chongqing Medical University, Chongqing 400016, China; 8 Department of Obstetrics and Gynecology, The Second Affiliated Hospital of Chongqing Medical University, Chongqing 400010, China

**Keywords:** quantitative metabolomics, stable isotope tracing, oral squamous cell carcinoma cells, metastasis

## Abstract

Most current metabolomics studies of oral squamous cell carcinoma (OSCC) are mainly focused on identifying potential biomarkers for early screening and diagnosis, while few studies have investigated the metabolic profiles promoting metastasis. In this study, we aimed to explore the altered metabolic pathways associated with metastasis of OSCC. Here, we identified four OSCC cell models (CAL27, HN6, HSC-3, SAS) that possess different invasive heterogeneity via the transwell invasion assay and divided them into high-invasive (HN6, SAS) and low-invasive (CAL27, HSC-3) cells. Quantitative analysis and stable isotope tracing using [U-^13^C_6_] glucose were performed to detect the altered metabolites in high-invasive OSCC cells, low-invasive OSCC cells and normal human oral keratinocytes (HOK). The metabolic changes in the high-invasive and low-invasive cells included elevated glycolysis, increased fatty acid metabolism and an impaired TCA cycle compared with HOK. Moreover, pathway analysis demonstrated significant differences in fatty acid biosynthesis; arachidonic acid (AA) metabolism; and glycine, serine and threonine metabolism between the high-invasive and low-invasive cells. Furthermore, the high-invasive cells displayed a significant increase in the percentages of ^13^C-glycine, ^13^C-palmitate, ^13^C-stearic acid, ^13^C-oleic acid, ^13^C-AA and estimated FADS1/2 activities compared with the low-invasive cells. Overall, this exploratory study suggested that the metabolic differences related to the metastatic phenotypes of OSCC cells were concentrated in glycine metabolism, de novo fatty acid synthesis and polyunsaturated fatty acid (PUFA) metabolism, providing a comprehensive understanding of the metabolic alterations and a basis for studying related molecular mechanisms in metastatic OSCC cells.

## 1. Introduction

Oral squamous cell carcinoma (OSCC) is the most common type of head and neck squamous cell carcinoma with approximately 600,000 new cases diagnosed per year worldwide [1,2]. OSCC is an aggressive cancer that can metastasize to the neck lymph nodes. And the median time to death is only 3.3 months once the tumors develop distant metastasis [3]. At present, researchers do not clearly understand the potential molecular mechanisms of OSCC metastasis, which leads to failure of treatment. Thus, it is necessary to investigate the comprehensive metabolic alterations involved in the malignant transformation and metastasis of OSCC and corresponding therapeutic targets.

Metabolic reprogramming is important for cancer cells to adapt to environmental pressures during initiation, proliferation and metastasis of cancer [4,5]. In recent years, a majority of metabolomics studies have focused on identifying potential biomarkers, exploring disease mechanisms, predicting the prognosis of cancer and evaluating therapeutic efficacy [6,7,8], while most metabolomics studies of OSCC have been applied to identify potential biomarkers for screening and diagnosis [9]. And some research detected 25 altered amino acids in OSCC tissues and further proved that L-asparagine promoted perineural invasion of OSCC [10]. Furthermore, it was reported that major metabolic variations associated with cancer metastasis appeared to involve pathways of lipid metabolism in OSCC [11,12]. Although these studies provide useful clues to metabolic dysfunctions, there is no comprehensive investigation of the metabolic profiles associated with metastasis in OSCC.

The invasiveness of cancer cells can be affected by the different origins, tissue types and genotypes of tumors. It is reflected not only in the different cell types (tumoral heterogeneity), the nature of the tumor–stroma interactions at the front of invasion and the role of growth factors and other substances that affect the invading cells [13,14,15] but also in the heterogeneity of cellular metabolism [12,16]. Tumoral heterogeneity and its associated alterations in metabolism are key factors for the resistance to treatment and metastasis formation [12,15], wherein intra-tumoral heterogeneity within cancer cells has been often employed to explore the metastatic mechanisms [17,18]. However, inter-tumoral heterogeneity is also very important in tumor metastasis [19,20]. Moreover, the microenvironment is variable inside the solid tumor with fluctuating levels of growth factors, oxygen and nutrients [21,22], which suggests that metastasis is complex. In order not to be affected by the complexities of the tumor microenvironment, the metabolic study of cancer cell lines can provide an advantage in exploring molecular alterations in metastatic cells. Thus, we envisage cancer cell lines with different metastatic heterogeneity as a subpopulation of cells, which were selected according to the background of cell line establishment.

Metabolomics, which can detect and analyze the precursors, intermediates and products of metabolic pathways, is often used to recognize the phenotypic state of a cell [23,24,25]. In addition, stable isotope-tracing techniques can examine intracellular fluxes, which aid the understanding of nutrient utilization and metabolic pathway activities [26]. Several research studies reported that multiple metabolic pathways were identified that are activated in cancer cells using isotopic tracer studies, including aerobic glycolysis [27], reductive metabolism of glutamine [28], serine and glycine metabolism and acetate metabolism [29]. In addition, multiple labeled substrates, such as ^13^C-glucose and ^15^N-glutamine, have been used to reveal metabolic alterations during tumorigenesis. The activities of metabolic pathways (pyruvate carboxylation) were associated with metastasis in breast cancer using ^13^C-tracer [30]. However, there is no single study integrating quantitative metabolomics and metabolic tracing of ^13^C-glucose carbons in metastatic OSCC cells.

In the present study, we collected some clinical samples and preliminarily analyzed the correlation between metabolite concentrations and TNM stage. Then, further exploration of the metabolic alterations related to metastasis was conducted in cell models. Four OSCC cell models with different invasive abilities were determined by taking advantage of tumoral heterogeneity and grouped into high-invasive and low-invasive cells. And we carried out quantitative GC–MS-based metabolomics and stable isotope tracing of high-invasive and low-invasive OSCC cells to confirm the altered metabolites and metabolic fluxes during metastasis, including glucose, amino acids, TCA intermediates and fatty acids.

## 2. Materials and Methods

### 2.1. Patients and Sample Collection

This study was ethically approved by the Research Ethics Committee of Stomatological Hospital of Chongqing Medical University, China (Project No. CQHS-REC-2021 (LSNo. 21), approved on 28 June 2021) following the Declaration of Helsinki. All participants included in this study signed the informed consent forms before enrolment. Patients with HPV infection, a history of chemotherapy and radiotherapy, oral metastasis of systemic tumors and other serious systemic diseases were excluded. The final diagnoses of those participants were confirmed with postoperative pathological examination, and these patients were staged according to the guidelines of AJCC8. Human OSCC and normal tissues were obtained immediately after surgery and snap-frozen in liquid nitrogen within 30 min. And then, the tissues were stored at −80 °C until further use. Table 1 shows the clinical characteristics of these patients.

### 2.2. Cell Culture

Four human OSCC cell lines, including SAS, HSC-3, CAL27 and HN6 [31,32,33,34], and one control cell line of normal human oral keratinocytes (HOK) were used. The SAS, HSC-3 and CAL27 cell lines were purchased from Cellbook Biotechnology Company (Guangzhou, Guangdong, China). The HN6 cell line was obtained from Dr. Hongying Chen (Chongqing Medical University, Chongqing, China). The HOK cell line was purchased from Tongpai Biotechnology Company (Shanghai, China). The OSCC cells were incubated in DMEM/F12 medium (Thermo Fisher Scientific, Waltham, MA, USA) supplemented with 10% fetal bovine serum (ExCell, Shanghai, China) and 1% streptomycin and penicillin (Hyclone, Logan, UT, USA). The HOK cell line was incubated in oral keratinocyte medium (Sciencell, Carlsbad, CA, USA). And all cells were incubated at 37 °C in a humidified 5% CO_2_ atmosphere. Then, all cells were cultured in DMEM/F12 medium with 10% fetal bovine serum when the cells were plated at 30% confluence before metabolite extraction in order to avoid the influence of different medium on these cells. And all these cells (three biological replicates for independent cell lines) were incubated for 36 h and extracted.

### 2.3. Transwell Invasion Assay

The Matrigel-based invasion assay was applied to characterize the invasive behavior of the OSCC cell lines [18]. The invasion assays of the SAS, HSC-3, CAL27 and HN6 cells were performed using 24-well plates and 8 μm transwell inserts (Thermo Fisher Scientific, Waltham, MA, USA), which were pre-coated with Matrigel (70 μL/well) (BD Biosciences, Bedford, MA, USA). A total of 2.5 × 10^5^ OSCC cells were suspended in 500 μL of serum-free DMEM/F12 medium and seeded in the upper chamber. And 700 μL of DMEM/F12 containing 10% FBS was added into the lower chambers. After incubation for 24 h, the cells in the upper chamber were removed using moistened cotton swabs, and the membranes were fixed in 4% paraformaldehyde for 15 min. The cells that migrated to the lower surface of the membrane were then stained with 0.1% crystal violet (Beyotime, Shanghai, China). We used microscopy (EVOS FL Auto Imaging System, Life Technologies, Carlsbad, CA, USA) to observe the cells on the lower chamber. Crystal violet was eluted in 600 μL of 33% acetic acid in each insert, and the absorbance at 570 nm was measured using SpectraMax iD5 (Molecular Devices, Sunnyvale, CA, USA). This experiment was repeated three times.

### 2.4. Metabolite Extraction

The tissue samples were prepared by dissecting 30.00 ± 0.50 mg into new tubes. And the tissues were homogenized via a TissueLyser II (QIAGEN, Dusseldorf, Germany) and centrifuged at 10,000× *g* for 15 min at 4 °C to isolate the supernatants after adding internal standards and 400 μL cold methanol. As for the cell culture, 500 μL of each culture medium was collected and used for chemical derivatization. Then, the cells were briefly rinsed thrice with cold 1× PBS. A total of 20 mL of liquid nitrogen was added to each flask of cells. Then, a cold mixture of methanol/sodium hydroxide (1:1) containing the internal standard d4-alanine (0.24 mM) was used to extract the metabolites from all the cells. The collected samples were centrifuged at 14,000× *g* for 15 min at 4 °C, and the supernatant was obtained. The supernatants were then stored at −80 °C for intracellular chemical derivatization.

### 2.5. Methyl Chloroformate (MCF) Derivatization and Gas Chromatography–Mass Spectrometry (GC–MS) Analysis

All the tissue samples and intracellular and extracellular samples were derivatized through the MCF derivatization, which was published in Nature protocols [35]. The chemical derivatives of the samples were analyzed with GC–MS (Agilent 5977A coupled to 7890B, Agilent technology, Santa Clara, CA, USA) with electron impact ionization set at 70 eV. The ZB-1701 GC capillary column (30 m × 250 μm id × 0.15 μm with 5 m guard column, Phenomenex, Los Angeles, CA, USA) was utilized to separate the chemical derivatives. Analysis of the GC–MS parameters was according to the procedure published previously [35].

### 2.6. GC–MS Data Processing and Targeted Quantitative Analysis

To identify the metabolites, the compounds were obtained by matching the in-house MCF library spectra > 85% and their corresponding GC retention time being within a window of 30 s. We utilized a commercial NIST mass spectral library to confirm any other unidentified compounds. The MassOmics R-based script was applied to extract the relative concentration of metabolites through the peak height of the most enriched fragmented iron mass within a specific retention time. The subtraction of background contamination and carryover from the identified metabolites was conducted using blank samples. With the aim to enhance quantitative reliability, the relative concentration of the identified metabolites was initially normalized using the internal standard (d4-alanine) based on their correlation with the metabolites in the quality control samples [36]. To address daily batch effects, median centering of the quality control samples was performed. The correction was achieved by weight in the tissue samples, while protein weight was applied for all the cell samples.

Chemical standards were employed for the quantification of amino acids, lactic acid and fatty acids. The concentrations of these metabolites were first normalized using the appropriate internal standard and then quantified to absolute concentration using calibration curves. Calibration curves were obtained from the respective chemical standard, spanning a concentration range of 0~195.8 μM.

### 2.7. [U-^13^C_6_] Glucose Labeling Experiment

For the labeling experiment, no-glucose DMEM/F12 medium (Procell, Wuhan, China) supplemented with 100% [U-^13^C_6_] glucose (Cambridge Isotope Laboratories, Tewksbury, MA, USA) was used for all cell cultures. As controls, the cells were cultured in an unlabeled DMEM/F12 medium with the same concentration of ^12^C_6_-glucose. The cells were seeded in ^13^C isotope-labeled or unlabeled medium into a 75 cm^2^ flask at 30% confluence. The cells were incubated in labeled medium for 36 h. The extracellular supernatants were then collected for measuring glucose and other metabolite levels. For all the cells, three independent biological replicates were performed, and separate identical 75 cm^2^ flasks were utilized for the protein assay.

### 2.8. Protein Quantification

Protein assays of the cell pellets were performed according to the protocol of the Beyotime BCA kit (Beyotime, Shanghai, China). The absorbance was measured on a spectrophotometer (SpectraMax iD5, Molecular Devices, Sunnyvale, CA, USA) at a wavelength of 562 nm after 30 min.

### 2.9. Glucose Assay

Glucose assays of the cell culture supernatants were performed using the Beyotime Glucose assay kit (Beyotime, Shanghai, China). In brief, 5 μL sample and 185 μL Glucose Assay Reagent were added to a PCR tube and heated at 95 °C for 8 min. The sample mixture was then cooled to 4 °C, and 180 μL liquid was transferred to a 96-well plate. The absorbance was measured at 630 nm with a spectrophotometer (SpectraMax iD5, Molecular Devices, Sunnyvale, CA, USA). Glucose quantification was calculated according to the standard curve established for each experiment. The glucose consumption was normalized by cell protein weight.

### 2.10. Statistical Analysis

To set up Gaussian distribution, the relative concentration of each metabolite was transformed with a log10 scale and Pareto scaling for this data. The metabolomic statistical analyses were conducted using MetaboAnalyst 5.0 (https://www.metaboanalyst.ca/ (accessed on 15 July 2023)). Multivariate analyses, including unsupervised principal component analysis (PCA), were performed to compare all cancer cell lines and HOK. And partial least squares discriminant analysis (PLS-DA) was performed to compare the high-invasive, low-invasive and normal cells. PLS-DA was validated through cross-validation and determined the variable importance in projection (VIP) to analyze the optimal number of the metabolites. The metabolic differences between the samples (high-invasive cells, low-invasive cells and normal cells) were analyzed using one-way ANOVA and Tukey’s honest significance difference (HSD) post hoc tests. A *p*-value < 0.05 and corresponding FDR < 0.05 were considered statistically significant. Hierarchical cluster analysis and pathway analysis were also conducted using MetaboAnalyst 5.0.

The metabolite ratios of tumor tissue/normal tissue were compared with the Mann–Whitney U test or Student’s *t*-test to detect significant differences in the metastasis (stage III and IV) and non-metastasis (stage I and II) groups. For the labeling experiments, the isotopic enrichments were calculated after natural isotope abundance correction. Comparisons of two groups were run using Student’s *t*-test. Multiple comparisons were analyzed using one-way ANOVA. Statistical analyses were performed using GraphPad Prism 8 software (GraphPad Co., Ltd., San Diego, CA, USA). *p* < 0.05 was considered statistically significant.

## 3. Results

### 3.1. Tumor Tissue/Normal Tissue Ratios of Metabolites versus Clinical Stage

There were fourteen amino acids (Table 2), six TCA cycle intermediates (Table 3) and twenty-one fatty acids (Table 4) identified in these tumor and normal tissues. To better reflect the changes in metabolite levels, we calculated the metabolite ratios of tumor tissue/normal tissue and compared them with clinical stage (metastasis (stage III and IV) and non-metastasis (stage I and II)). The analysis demonstrated that glycine, proline, docosapentaenoic acid (DPA), docosahexaenoic acid (DHA), linoleic acid (LA), gamma-linolenic acid (GLA), dihomo-gamma-linolenic acid (DGLA) and arachidonic acid (AA) were significantly associated with metastatic stages of OSCC, whereas no other metabolites were significantly related to the clinical stages of OSCC.

### 3.2. Establishing the Invasive Capacity of OSCC Cell Lines

**Figure 1:**
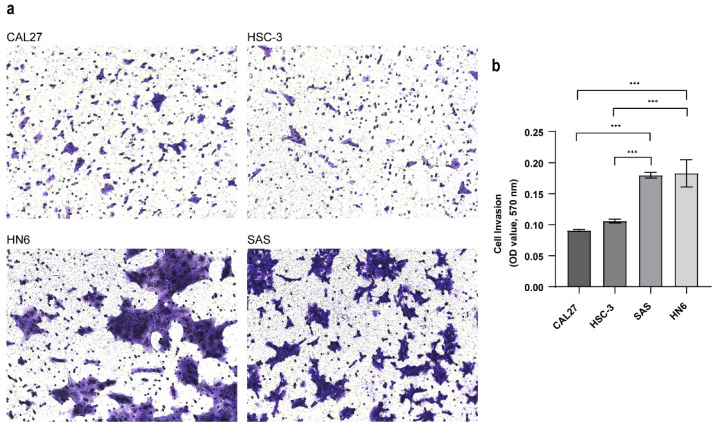
Transwell invasion assay. (**a**) Cell invasion ability was evaluated with the transwell assay (mag. ×200). Pictures were taken after 24 h. (**b**) The absorbance was measured at 570 nm. Data were analyzed using one-way ANOVA and represented as mean ± SD (*** *p* < 0.001).

Whether these different metabolites associated with metastatic OSCC are derived from cancer cells remains unclear because the components of the tumor microenvironment include local stromal cells (resident fibroblasts and macrophages) and distant recruited cells (endothelial cells, immune cells). Thus, we next evaluated the metabolic changes in the OSCC cell lines with different metastatic capacity and explored the molecular mechanisms associated with metastasis. To confirm the invasive abilities of the OSCC cell lines in vitro, we performed a Matrigel-based invasion assay. In this study, there were four OSCC cell lines analyzed, including CAL27, HN6, HSC-3 and SAS. The invasion rate of HN6 was similar to that of SAS, and the invasion rates of Cal27 and HSC3 were also similar (Figure 1a), while the invasiveness of HN6 and SAS was significantly higher than that of CAL27 and HSC-3 (Figure 1b). Thus, we categorized the CAL27 and HSC-3 cells into low-invasive cancer cells and HN6 and SAS cells into high-invasive cancer cells.

### 3.3. Metabolic Profiling Alterations of Normal Cells and Low-Invasive and High-Invasive Cancer Cells Based on Untargeted Metabolomics

**Figure 2:**
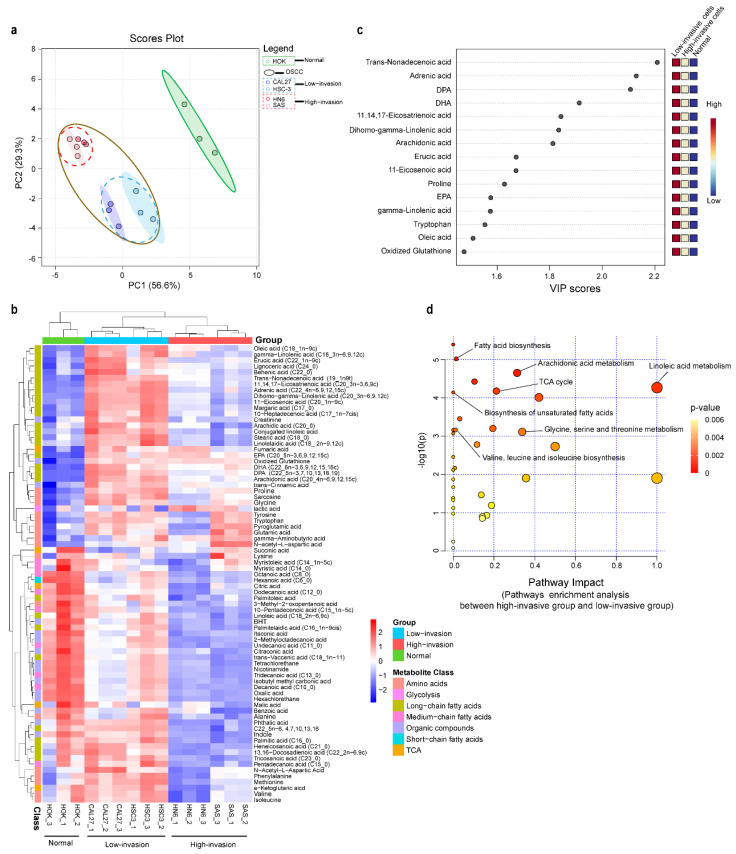
Metabolomic profiles of high-invasive cancer cells, low-invasive cancer cells and normal cells. (**a**) Principal component analysis (PCA) of metabolites demonstrated separation of high-invasive cells, low-invasive cells and normal cells. The mass spectrometry abundance of each cell type with three independent biological replicates clustered together. Each point in the plot represents a cell sample. Red dots represent HN6, pink dots represent SAS, blue dots represent HSC-3, dark blue dots represent CAL27, and green dots represent HOK; blue-dotted circle represents low-invasive cancer cells, red-dotted circle represents high-invasive cancer cells, and brown circle represents all OSCC cells. (**b**) The heatmap shows metabolic profiles in high-invasive cells, low-invasive cells and normal cells, which was produced with a hierarchical clustering algorithm using elucidation distance. (**c**) VIP scores of the top 15 significantly altered metabolites that were important for high-invasive cells, low-invasive cells and normal cells with partial least squares discriminant analysis (PLS-DA) (VIP > 1, *p* < 0.05, q < 0.05). (**d**) Pathway analysis of metabolites found dysregulated in high-invasive cells compared with low-invasive cells.

A total of 92 metabolites were identified in the high-invasive and low-invasive cancer cells and normal cells. To compare the metabolite compositions, unsupervised principal component analysis (PCA) was performed. In the PCA plot (Figure 2a), similar characteristics within each cell type were demonstrated, indicating the reproducibility of the metabolic profile of three independent biological replicates. Also, the PCA plot and the hierarchical clustering heatmap showed that the high-invasive cells and low-invasive cells clustered together, respectively, and both of them were clearly differentiated from the normal cells (Figure 2a,b). Different metabolomic profiles between the normal cells and the low-invasive and high-invasive cells were obtained with partial least squares discriminant analysis (PLS-DA) and VIP scores. The top fifteen discriminating metabolites are demonstrated according to the VIP scores in Figure 2c, including DPA, DHA, AA, DGLA, eicosapentaenoic acid (EPA) and so on (VIP > 1, *p* < 0.05, q < 0.05). These fatty acids were informative to distinguish between the high- and low-invasive cells and normal cells. To further demonstrate the potential molecular mechanism in the high-invasive and low-invasive cancer cells, the metabolic pathways of the metabolites were analyzed with MetaboAnalyst (Figure 2d). The results showed that arachidonic acid metabolism; linoleic acid metabolism; fatty acid biosynthesis; biosynthesis of unsaturated fatty acids; and glycine, serine and threonine metabolism were associated with the invasion of OSCC cells.

### 3.4. The Different Concentration of Glucose, TCA Cycle Intermediates and Amino Acids between High-Invasive and Low-Invasive Cancer Cells

**Figure 3:**
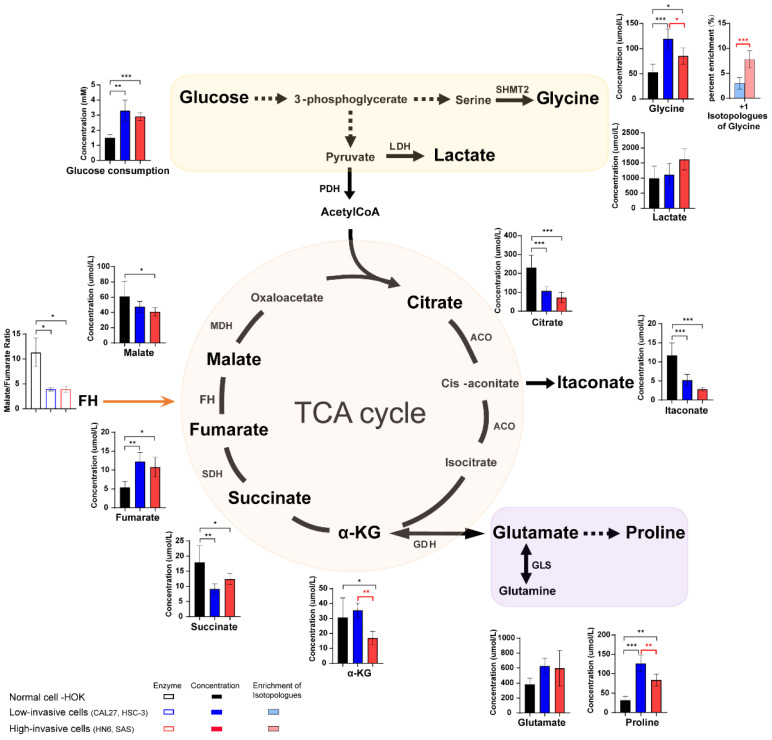
The schematic shows the metabolic pathways (including glycolysis, some amino acids and TCA cycle) in the cells, and the bar plots illustrate the metabolite levels detected in the high-invasive cells (HN6, SAS), low-invasive cells (CAL27, HSC-3) and normal cells. Bar plots demonstrate absolute concentrations of lactate, TCA-cycle intermediates and amino acids. And box bar plots show estimated FH activity (the ratio of malate/fumarate). Black represents normal cells, blue represents low-invasive cells, and red represents high-invasive cells. Pink and light blue bar plots show the enrichment fractions of ^13^C-glycine from [U-^13^C_6_] glucose. Black asterisks (*) indicate a significant difference in metabolites and enzymic activities between the high-invasive cells and normal cells and between the low-invasive cells and normal cells. Red asterisks (*) indicate a significant difference in metabolites, enzymic activities and ^13^C enrichment between the high-invasive cells and low-invasive cells. Comparisons of two groups were run using Student’s *t*-test. Multiple comparisons were analyzed using one-way ANOVA and represented as mean ± SD (* *p* < 0.05; ** *p* < 0.01; *** *p* < 0.001). SHMT2: serine hydroxyl-methyltransferase 2; LDH: lactate dehydrogenase; PDH: pyruvate dehydrogenase complex; ACO: aconitase; SDH: succinate dehydrogenase; FH: fumarate hydratase; MDH: malate dehydrogenase; α-KG: alpha-ketoglutarate; GDH: glutamate dehydrogenase; GLS: glutaminase.

In order to further determine the changes in the concentrations of metabolites and analyze metabolic reprogramming of OSCC cells better, absolute quantification of glucose, TCA-cycle intermediates, fatty acids, lactate and amino acids was performed on the high-invasive and low-invasive cancer cells and HOK. It was observed that the concentrations of glucose uptake, glycine and proline were increased, the levels of TCA-cycle intermediates (except fumarate levels) were elevated, and estimated FH activities (fumarate hydratase, the ratio of malate/fumarate) were reduced in the cancer cells compared with the normal cells (Figure 3). In addition, the levels of glycine, as a byproduct of glycolysis, were significantly lower, but the fraction of ^13^C-glycine was higher in the high-invasive cancer cells compared with those in the low-invasive cancer cells. And proline, valine, methionine, phenylalanine and isoleucine levels were significantly higher in the low-invasive cells than those in the high-invasive cells (Supplementary Figure S1). However, there was no significant difference in the levels of glucose intake, lactate, TCA-cycle metabolites (except alpha-ketoglutarate (α-KG)) and glutamate and the enrichment fractions of most amino acids (proline, glutamate, tyrosine, valine, methionine, lysine, phenylalanine and isoleucine) in the high-invasive and low-invasive cancer cell comparison (Figure 3, Supplementary Figure S1).

### 3.5. Long-Chain Fatty Acid Metabolism between High-Invasive and Low-Invasive Cancer Cells

**Figure 4:**
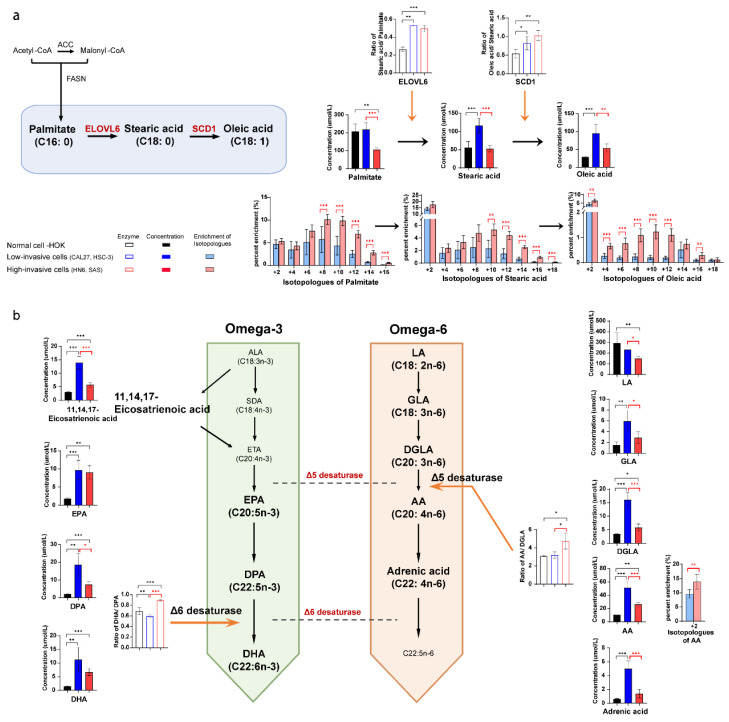
The intracellular long-chain fatty acid metabolic differences between the high-invasive cells (HN6, SAS), low-invasive cells (CAL27, HSC-3) and normal cells. (**a**) The diagram demonstrates the main metabolic pathways of de novo fatty acid synthesis in cancer cells. Bar plots demonstrate absolute concentrations of palmitate, stearic acid and oleic acid. And box bar plots show estimated SCD1 activity and ELOVL6 activity. Black represents normal cells, blue represents low-invasive cells, and red represents high-invasive cells. Pink and light blue bar plots show the enrichment fractions of ^13^C-palmitate, ^13^C-stearic acid and ^13^C-oleic acid from [U-^13^C_6_] glucose. (**b**) The schematic shows the metabolic pathways of omega-3 and omega-6 polyunsaturated fatty acids (PUFA) in cancer cells. Bar plots demonstrate absolute concentrations of omega-3 and omega-6 PUFA. And box bar plots show estimated delta-5 and delta-6 desaturase activities. Black represents normal cells, blue represents low-invasive cells, and red represents high-invasive cells. Pink and light blue bar plots show the enrichment fractions of ^13^C-AA from [U-^13^C_6_] glucose. Black asterisks (*) indicate a significant difference in metabolites and enzymic activities between the high-invasive cells and normal cells and between the low-invasive cells and normal cells. Red asterisks (*) indicate a significant difference in metabolites, enzymic activities and ^13^C enrichment between the high-invasive cells and low-invasive cells. Comparisons of two groups were run using Student’s *t*-test. Multiple comparisons were analyzed using one-way ANOVA and represented as mean ± SD (* *p* < 0.05; ** *p* < 0.01; *** *p* < 0.001). FASN: fatty acid synthase; ACC: acetyl-CoA carboxylase; ALA: alpha-linolenic acid; SDA: stearidonic acid; ETA: eicosatetraenoic acid; LA: linoleic acid; DGLA: dihomo-gamma-linolenic acid; EPA: eicosapentaenoic acid; DPA: docosapentaenoic acid; DHA: docosahexaenoic acid. AA: arachidonic acid; GLA: gamma-linolenic acid; SCD1: stearoyl-CoA desaturase 1, oleic acid/stearic acid (C18:1/C18:0); ELOVL6: elongase 6, stearic acid/palmitate (C18:0/C16:0); delta-6 desaturase: FADS2, DHA/DPA (C22:6n-3/C22:5n-3); delta-5 desaturase: FADS1, AA/DGLA (C20:4n-6/C20:3n-6).

Healthy human cells use exogenous fatty acids for intracellular lipid metabolism, while tumor cells mainly use endogenous fatty acids through up-regulation of de novo fatty acid synthesis. In order to explore the metabolic changes derived from glucose, we compared the difference in intracellular fatty acids and ^13^C-fatty acids between the high-invasive cells, low-invasive cells and normal cells. The concentrations of almost all omega-3 and omega-6 fatty acids increased, and the estimated SCD1 (stearoyl-CoA desaturase 1, ratio of oleic acid/stearic acid) and ELOVL6 (ratio of stearic acid/palmitate) activities were upregulated in the OSCC cells compared with the normal cells (Figure 4a,b). Wherein, AA had the biggest increases in both the high-invasive and low-invasive cells. Interestingly, we found that the high-invasive cells had a lower concentration of de novo synthesized fatty acids (palmitate, stearic acid and oleic acid) and a higher enrichment of ^13^C-palmitate, ^13^C-stearic acid and ^13^C-oleic acid compared with the low-invasive cells (Figure 4a). Furthermore, most omega-3 and omega-6 fatty acids showed a significant increase in concentrations in the low-invasive cells. However, the high-invasive cells had elevated estimated activities of delta-5 desaturase (FADS1, ratio of C20:4n-6/C20:3n-6) and delta-6 desaturase (FADS2, ratio of C22:6n-3/C22:5n-3) and an increased faction of ^13^C-AA (Figure 4b).

### 3.6. ^13^C-Enrichment of Metabolites Derived from Glucose in Normal Cells and High-Invasive and Low-Invasive Cells

**Figure 5:**
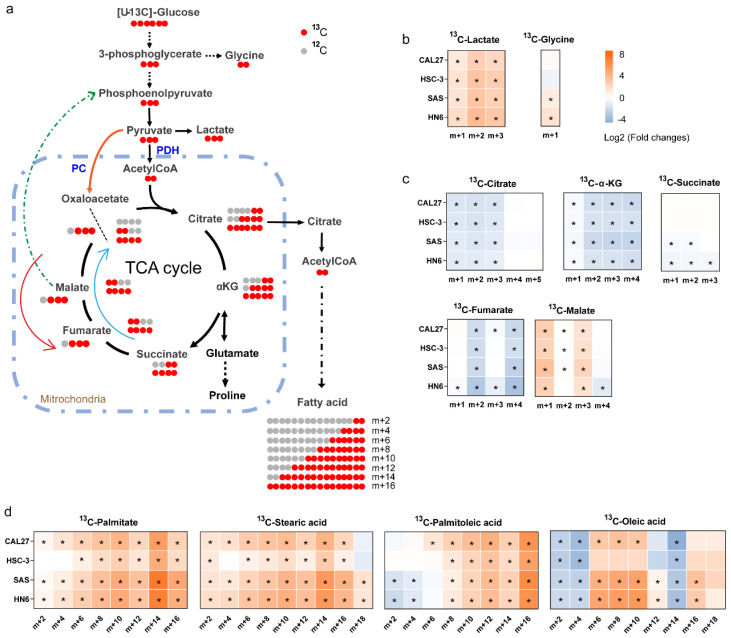
[U-^13^C_6_] glucose tracing revealed the flow of carbon atoms in the high-invasive and low-invasive cells. (**a**) Schematic diagram shows labeling patterns of downstream metabolites following uptake of [U-^13^C_6_] glucose. The orange arrow shows ^13^C-pyruvate enters the TCA cycle via the pyruvate carboxylase reaction. The green-dotted arrow represents that possible ^13^C-isotopologues derived from ^13^C-oxaloacetate are used for gluconeogenesis through a series of enzymatic reactions. The blue arrow represents the oxidative TCA cycle, and the red arrow shows the reductive TCA cycle. (**b**) The heatmaps show the ratios of ^13^C isotopologues fraction of glycolytic intermediates, including lactate (m+1, m+2, m+3) and glycine (m+1), between each OSCC cell and HOK. (**c**) The heatmaps show the ratios of ^13^C isotopologues fraction of the TCA-cycle intermediates between each OSCC cell and HOK; the plots only demonstrate ^13^C isotopologues detected in our experiment, including ^13^C-citrate (m+1, m+2, m+3, m+4, m+5), ^13^C-α-KG (m+1, m+2, m+3, m+4), ^13^C-succinate (m+1, m+2, m+3), ^13^C-fumarate (m+1, m+2, m+3, m+4) and ^13^C-malate (m+1, m+2, m+3, m+4). (**d**) The heatmaps show the ratios of ^13^C isotopologues fraction of de novo synthesized fatty acids, including palmitate, palmitoleic acid, stearic acid and oleic acid. The relative distributions of ^13^C isotopologues of metabolites are demonstrated using log2 scale. Red color represents a higher ^13^C isotopologue fraction in the OSCC cells than that in HOK, and blue color indicates a lower ^13^C isotopologue fraction in the OSCC cells than that in HOK. * *p* < 0.05. PDH: pyruvate dehydrogenase complex; PC: pyruvate carboxylase.

To further understand the difference in cell metabolism derived from glucose between the OSCC cells and HOK, we conducted ex vivo stable isotope labeling using [U-^13^C_6_] glucose as the only carbon source (Figure 5a). The labeled carbon atoms of the metabolites were increased, demonstrating the conversion of labeled carbon into metabolites. In a word, the ^13^C labeled metabolites exhibit higher biochemical conversion rates. As we can observe, lactate demonstrated a higher rate of conversion in the high-invasive and low-invasive cells compared with HOK (Figure 5b), indicating an increase in glycolysis, wherein the percent of m+2 lactate increased more than that of m+3 lactate, which suggested glycolysis was not the only source of lactate. In addition, ^13^C-citrate, ^13^C-α-KG and ^13^C-fumarate were contrary in the high-invasive and low-invasive cells (Figure 5c), which meant the inactivation of the pyruvate dehydrogenase complex (PDH) and TCA-cycle dysfunction in the cancer cells. Interestingly, the fraction of m+3 malate was approximately three-fold higher in the high-invasive and low-invasive cells (Figure 5c), which indicated m+3 pyruvate entered the TCA cycle through carboxylation by PC, which is called pyruvate anaplerosis. Furthermore, the conversion rates of the labeled de novo synthesized fatty acids (^13^C-palmitate, ^13^C-palmitoleic, ^13^C-stearic acid and ^13^C-oleic acid) were significantly increased in both the high-invasive and low-invasive cells (Figure 5d).

### 3.7. Concentration of Extracellular Metabolites between High-Invasive and Low-Invasive Cancer Cells

**Figure 6:**
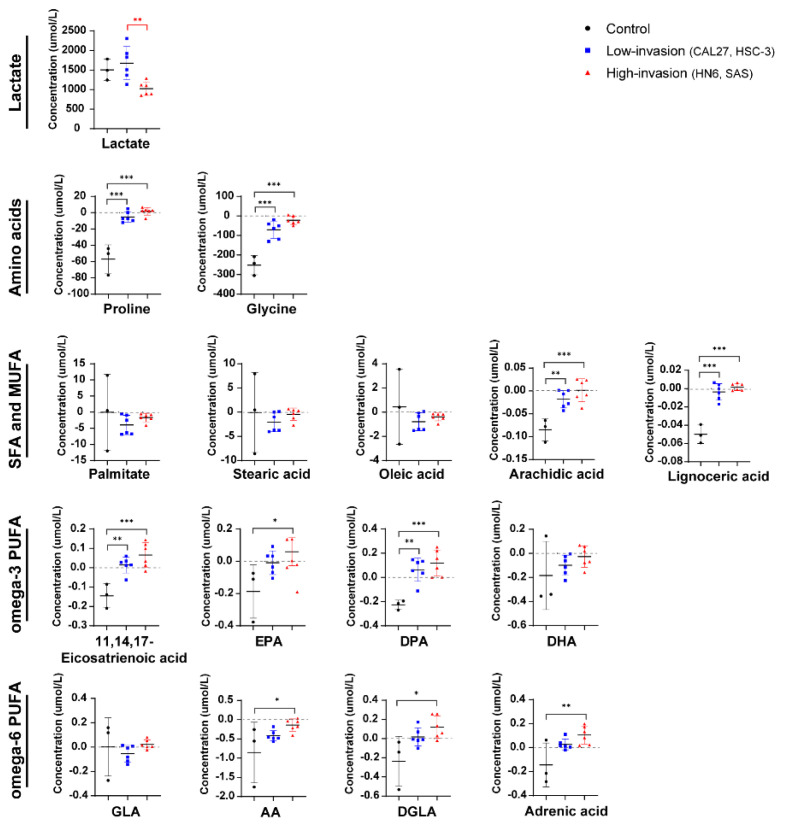
Extracellular metabolite difference between the high-invasive cells (HN6, SAS), low-invasive cells (CAL27, HSC-3) and HOK. The plots show the metabolite differences, including lactate, amino acids and fatty acids, in the extracellular medium between the high-invasive cells, low-invasive cells and HOK. Above the black line (positive values) means secretion, and below the black line (negative values) indicates absorption. Black asterisks (*) indicate a significant difference in metabolites between the high-invasive cells and normal cells and between the low-invasive cells and normal cells. Red asterisks (*) indicate a significant difference in metabolites between the high-invasive cells and low-invasive cells. Data are analyzed using one-way ANOVA and represented as mean ± SD (* *p* < 0.05; ** *p* < 0.01; *** *p* < 0.001).

We further examined the metabolite differences in the extracellular medium. The lactate levels of the OSCC cells were not significantly different from those of HOK (Figure 6), inconsistent with the results of intracellular lactate (Figure 3). And we observed that the OSCC cells promoted extracellular secretion or reduced extracellular absorption of the metabolic profiles of amino acids and fatty acids relative to the HOK cells (Figure 6). The results suggested that the OSCC cells preferred to utilize endogenous metabolites and release some of these metabolites into the medium. Especially, we found that the high-invasive cells released more DPA, EPA, DGLA, 11,14,17-eicosatrienoic acid and adrenic acid compared with the low-invasive cells, although not reaching statistical significance.

## 4. Discussion

This is a study to assess the comprehensive metabolic alterations of high-invasive and low-invasive OSCC cells using quantitative metabolomics and isotope-labeling experiments. We determined subpopulations of OSCC cells by taking advantage of metastatic variability and grouped them into high-invasive (HN6, SAS) and low-invasive (CAL27, HSC-3) cells. Our data demonstrated that the major variations between the high-invasive and low-invasive cancer cells involved pathways of glycine and fatty acid metabolism (de novo fatty acid synthesis and omega-3/omega-6 PUFAs). De novo synthesized fatty acids displayed higher conversion rates from [U-^13^C_6_] glucose and were incorporated into lipids in the high-invasive cells. In particular, the increase in estimated FADS1 and FADS2 activities could lead to altered intracellular levels and extracellular release of omega-3/omega-6 PUFAs, which could contribute to invasion of OSCC cells.

### 4.1. Metabolic Reprogramming of Low-Invasive and High-Invasive Cancer Cells Compared with Normal Cells

The metabolic reprogramming of OSCC cells promoted glucose utilization and glycolysis but impaired the TCA cycle, which meant the switch from oxidative phosphorylation to glycolysis in the OSCC cells. These were revealed by the increased levels of glucose uptake and glycolytic byproduct (^13^C-glycine, ^13^C-lactate) and the reduced enrichment fractions of the TCA intermediates (^13^C-citrate, ^13^C-α-KG, ^13^C-succinate, ^13^C-fumarate) in all OSCC cells. It was also reported that OSCC cells had an increased aerobic glycolysis, also called the Warburg effect, for the production of ATP as mitochondrial respiration exhibited abnormal functions [37,38,39]. However, only the concentration of fumarate in the TCA-cycle intermediates was significantly increased, and the estimated FH activity (the ratio of malate/fumarate) was significantly reduced in the OSCC cells in our study. Several researchers also reported increased fumarate levels in OSCC cells through untargeted metabolomics [38,40]. Shingo Noguchi et al. demonstrated that the diminished FH activity resulted in the decrease in TCA cycle flux and downregulation of ATP production derived from the TCA cycle [41]. It was also proposed that pyruvate dehydrogenase activity was inhibited, and the carbon atoms of glucose were restricted from entering the TCA cycle in FH-defect cells via ^13^C tracer analysis and phosphoproteome analysis [42]. Based on these findings, we suggested that the FH defect, leading to accumulation of fumarate, was associated with impaired mitochondrial function in the OSCC cells. Notably, the OSCC cells did not use glutamine to replenish the TCA cycle and possibly converted it to proline. In our study, the concentrations of α-KG and glutamate did not rise, and the level of proline was elevated in the OSCC cells. Similarly, Sandulache et al. reported that head and neck squamous cell carcinoma mainly relied on glucose, not glutamine, for energy production [43]. In short, glucose, not glutamine, was the main source of OSCC cell energy and provided energy through glycolysis, which compensated for the mitochondrial dysfunction caused by the diminished FH activity.

### 4.2. Metabolic Alterations between Low-Invasive Cancer Cells and High-Invasive Cancer Cells

We found that invasion of OSCC cells seemed to be related to high glycine conversion from glucose. Our data demonstrated that glycine metabolism was associated with invasion of cancer cells (Figure 2), and the enrichment of ^13^C-glycine was higher in the high-invasive cells. Increased glycine is a common metabolic adaptation in many cancers [29,44] because glycine drives one-carbon metabolism to the synthesis of nucleic acids, proteins and lipids utilized in cellular growth and proliferation. In addition, serine hydroxyl-methyltransferase 2 (SHMT2) transforms serine into glycine and functions as a crucial component for the serine/glycine metabolism in various cells. SHMT2 overexpression was reported to be related to breast cancer tumor aggressiveness in a dose-dependent manner [45]. Wu et al. confirmed that SHMT2 was linked to aggressive progression of gliomas, and SHMT2 knockdown inhibited the proliferation and invasion of glioma cells in vitro [46]. On the other hand, our results demonstrated that the high-invasive cells had lower glycine levels. It was proposed that the accumulation of glycine impaired cell viability after inhibition of glycine decarboxylase, which catalyzes the oxidative decarboxylation and deamination of glycine [47]. Another study showed that a high expression of glycine decarboxylase protein in primary melanoma was closely linked to early metastasis in patients [48]. Similar results were reported in non-small cell lung cancer [49]. Thus, these findings indicated that a high metabolic rate of glycine played an important role in high-invasive OSCC cells through SHMT2 and glycine decarboxylase.

In our study, de novo fatty acid synthesis appeared to be linked to metastatic phenotypes of the OSCC cells. The labelling of ^13^C-palmitate, ^13^C-stearic acid and ^13^C-oleic acid was higher in the high-invasive cells when glucose uptake was similar in the high-invasive and low-invasive cancer cells. Unexpectedly, the levels of these fatty acids were not higher in metastatic tumors and the high-invasive cells. It was reported that the accumulation of lysophosphatidylcholine (LPC) [16:0] was more pronounced in the area of healthy stroma near the aggressive front of the tumor, and LPC [16:0] promoted invasion in vitro in head and neck squamous cell carcinoma [50]. Moreover, some reports showed that de novo synthesized fatty acids derived from [U-^13^C_6_] glucose were incorporated into a variety of lipids, such as sphingolipids, glycerolipids and glycerophospholipids, in cancer cells [51]. And free fatty acid incorporation into oncogenic signaling lipids was elevated in high-invasive cancer cells compared with low-invasive cancer cells [52]. In addition, more de novo synthesized fatty acids were incorporated into the membrane phospholipids in breast cancers compared with healthy tissue, and this process was further increased during cancer progression [53]. Therefore, we suggested that high-invasive cells upregulated de novo fatty acid synthesis and rapidly incorporated newly synthesized fatty acids into complex lipids rather than existing in free fatty acid pools, which can promote metastasis in OSCC cells.

Interestingly, the extracellular release of omega-3 PUFAs was possibly involved in promoting invasion of OSCC cells. Our results showed an increase in the levels of intracellular and extracellular omega-3 PUFAs (11,14,17-eicosapentaenoic acid, EPA and DPA) in the cancer cells, which were suggested to be closely related to the conversion efficiency of their endogenous synthesis and release rather than extracellular absorption. Contrary to this, most published reports demonstrated that high levels of omega-3 PUFAs were related only to an increased intake, and they reduced tumor invasion and metastasis [54]. Cancer cell line culture, which is not affected by the tumor microenvironment and exogenous omega-3 PUFAs, might explain the inconsistency between previous research and our findings. Additionally, we found that intracellular omega-3 PUFA levels were lower, and extracellular omega-3 PUFAs were slightly higher in the high-invasive cells compared with the low-invasive cells, which indicated that the high-invasive cells released more omega-3 PUFAs. Higher levels of omega-3 PUFAs in our metastatic tumor tissues might be released from the cancer cells. Several researchers also found that omega-3 fatty acids were positively correlated with tumor grade in the serum of OSCC patients [55]. Furthermore, Brasky et al. showed that DHA was positively related to high-grade prostate cancer (OR, 2.50; 95% CI, 1.34–4.65) [56]. And omega-3 PUFAs incorporated into membrane phospholipids could be released by phospholipase A2 and other enzymes and converted into multiple oxylipins, which modulate proliferation, cell survival and invasion [57]. In addition, it was proposed that long-chain fatty acids were accumulated in the tumor microenvironment and limited CD8+ T cell infiltration and functions in pancreatic ductal adenocarcinoma [58]. Another study showed that the RHOA Y42 mutation induced abundant free fatty acid (including omega-3 PUFAs) production in cell line culture, which contributed to the immunosuppressive function of Treg cells that promoted progression of gastric cancer [59]. On these bases, we implied that the elevated endogenous synthesis and release of omega-3 PUFAs were related to invasion of OSCC cells by contributing to multiple oxylipins and the immunosuppressive tumor microenvironment. Future research on the role of individual omega-3 PUFAs in OSCC cells is needed.

AA metabolism can promote metastasis in OSCC cells. Our data showed the increased enrichment of ^13^C-AA and estimated FADS1 activity in the high-invasive cancer cells. The omega-6 PUFAs exert pro-inflammatory and pro-tumorigenic effects in cancer cells [60,61]. AA can produce prostaglandin E2 (PGE2) by COX-2. And PGE2 was reported to promote cancer migration via inducing MMP-9 expression [62]. Navarro-Tito illustrated that AA induced focal adhesion kinase phosphorylation and promoted metastasis [63]. Moreover, FADS1, which catalyzes DGLA to AA, was also increasingly expressed in esophageal squamous cell carcinoma and regulated cancer cell invasion through the Akt/mTOR pathway [64]. FADS1 knockdown significantly inhibited growth and metastasis in breast cancer [65]. In short, invasive phenotypes were associated with the production of AA by FADS1 and transformation of AA to PGE2 by COX-2.

## 5. Conclusions

**Figure 7:**
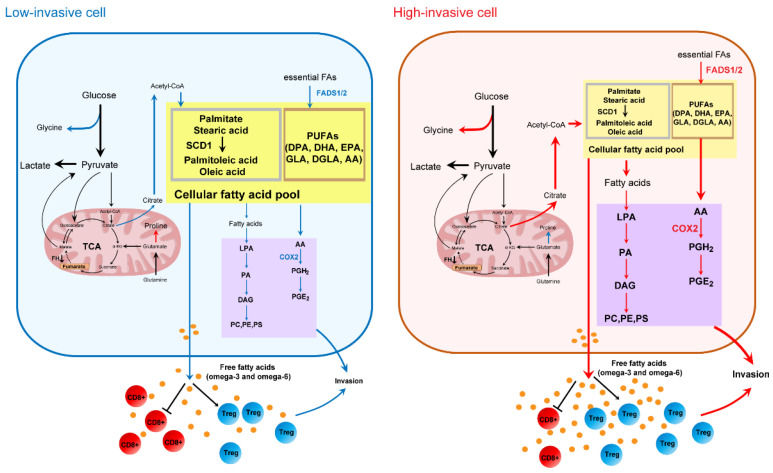
Metabolic differences between high-invasive and low-invasive OSCC cells. The color of the arrows and proteins notes high (in red), low (in blue) or unchanged (in black) fluxes or protein expression between high-invasive and low-invasive cells.

In summary, this is a pilot research attempt to explore the metabolic alterations between high-invasive (HN6, SAS) and low-invasive (CAL27, HSC-3) OSCC cell lines. Amino acid metabolism and fatty acid metabolism were prominently related to the invasiveness of OSCC cells, which involved several pathways connected to glycine metabolism, de novo fatty acid synthesis and omega-3/omega-6 PUFA metabolism (Figure 7). Overall, this study provides important information on the metabolic changes in metastatic cells and can aid the understanding of the molecular mechanisms associated with the metabolic pathways in metastatic OSCC cells. Future studies should use different tracers combined with cell and animal experiments to better understand the metastatic mechanisms related to cell metabolism.

## Figures and Tables

**Table 1:** Baseline characteristics of patients with OSCC.

Parameters	
Gender	12 males, 5 females
Age (y)	63 ± 9.5
TNM stage	No. of patients
I + II	9
III + IV	8
Tumor grade	
G1	9
G2	6
G3	2
Primary sites	
Buccal mucosa	3
Tongue	8
Gum	2
Floor of mouth	2
Hard palate	2

**Table 2:** Correlation of amino acid ratios of tumor tissue/normal tissue with tumor clinical status.

	Ratios of Tumor Tissue/ Normal Tissue		
Amino Acids	Stage I and II (Non-Metastatic)	Stage III and IV (Metastatic)	*p*-Value
Proline	4.51	1.90	0.036
Glycine	1.58	4.54	0.021
Leucine	1.74	3.24	0.200
Methionine	3.08	3.72	0.409
Alanine	1.13	1.22	0.412
Isoleucine	2.34	3.27	0.963
Serine	2.31	1.89	0.482
Glutamic acid	2.61	3.17	0.486
Phenylalanine	2.18	1.84	0.200
Threonine	2.21	2.38	0.788
Tryptophan	2.00	1.75	0.541
Tyrosine	1.96	1.83	0.888
Histidine	1.51	1.43	0.799
Lysine	1.65	1.56	0.822

**Table 3:** Correlation of TCA cycle intermediate ratios of tumor tissue/normal tissue with tumor clinical status.

	Ratios of Tumor Tissue/ Normal Tissue		
TCA Cycle	Stage I and II (Non-Metastatic)	Stage III and IV (Metastatic)	*p*-Value
Malate	1.17	1.69	0.075
α-Ketoglutarate	1.23	1.56	0.743
Succinate	3.04	1.92	0.139
Cis-aconitate	3.33	2.36	0.888
Itaconate	1.38	1.28	0.585
Fumarate	1.13	1.21	0.732

**Table 4:** Correlation of fatty acid ratios of tumor tissue/normal tissue with tumor clinical status.

	Ratios of Tumor Tissue/ Adjacent Normal Tumor Tissue		
Fatty Acids	Stage I and II (Non-Metastatic)	Stage III and IV (Metastatic)	*p*-Value
SFAs			
Decanoic acid (C10:0)	0.97	0.97	0.815
Dodecanoic acid (C12:0)	0.88	0.88	>0.999
Myristic acid (C14:0)	1.08	2.11	0.093
Palmitate (C16:0)	0.81	1.00	0.339
Stearate (C18:0)	2.78	2.99	0.423
Arachidic acid (C20:0)	1.20	1.36	0.541
Lignoceric acid (C24:0)	1.87	2.27	0.481
MUFAs			
Myristoleic acid (C14:1)	1.09	1.91	0.139
Oleic acid (C18:1)	0.87	1.38	0.093
Gondoic acid (C20:1)	0.99	1.84	0.073
Erucic acid (C22:1)	1.04	1.80	0.012
Nervonic acid (C24:1)	0.99	1.39	0.049
Omega-3 PUFAs			
11,14,17-Eicosatrienoic acid (C20:3n-3)	0.51	0.82	0.128
EPA (C20:5n-3)	1.33	2.27	0.101
DPA (C22:5n-3)	1.01	2.12	0.003
DHA (C22:6n-3)	1.13	2.32	0.004
Omega-6 PUFAs			
LA (C18:2n-6)	0.81	2.11	0.009
GLA (C18:3n-6)	0.41	1.84	0.003
11,14-Eicosadienoic acid (C20:2n-6)	1.70	1.84	0.746
DGLA (C20:3n-6)	0.97	1.85	0.034
AA (C22:4n-6)	1.01	2.37	0.002

EPA: eicosapentaenoic acid; DPA: docosapentaenoic acid; DHA: docosahexaenoic acid; LA: linoleic acid; GLA: gamma-linolenic acid; DGLA: dihomo-gamma-linolenic acid; AA: arachidonic acid.

## Data Availability

The datasets generated and analyzed during the current study are available from the corresponding author upon reasonable request.

## Supplementary Materials

The following supporting information can be downloaded at: https://www.mdpi.com/article/10.3390/biom13121806/s1, Figure S1: The intracellular amino acid metabolic differences between high-invasive cells and low-invasive cells.
