# Supplementary material for: Metabolic Patterns of High-Invasive and Low-Invasive Oral Squamous Cell Carcinoma Cells Using Quantitative Metabolomics and 13C-Glucose Tracing
Source: Biomolecules. 2023 Dec 18;13(12):1806. doi: 10.3390/biom13121806 (PMC10742159; doi:10.3390/biom13121806)
Supplement: Supplementary file 1 [file biomolecules-13-01806-s001.zip › Supplementary information.pdf]

Supplementary Information

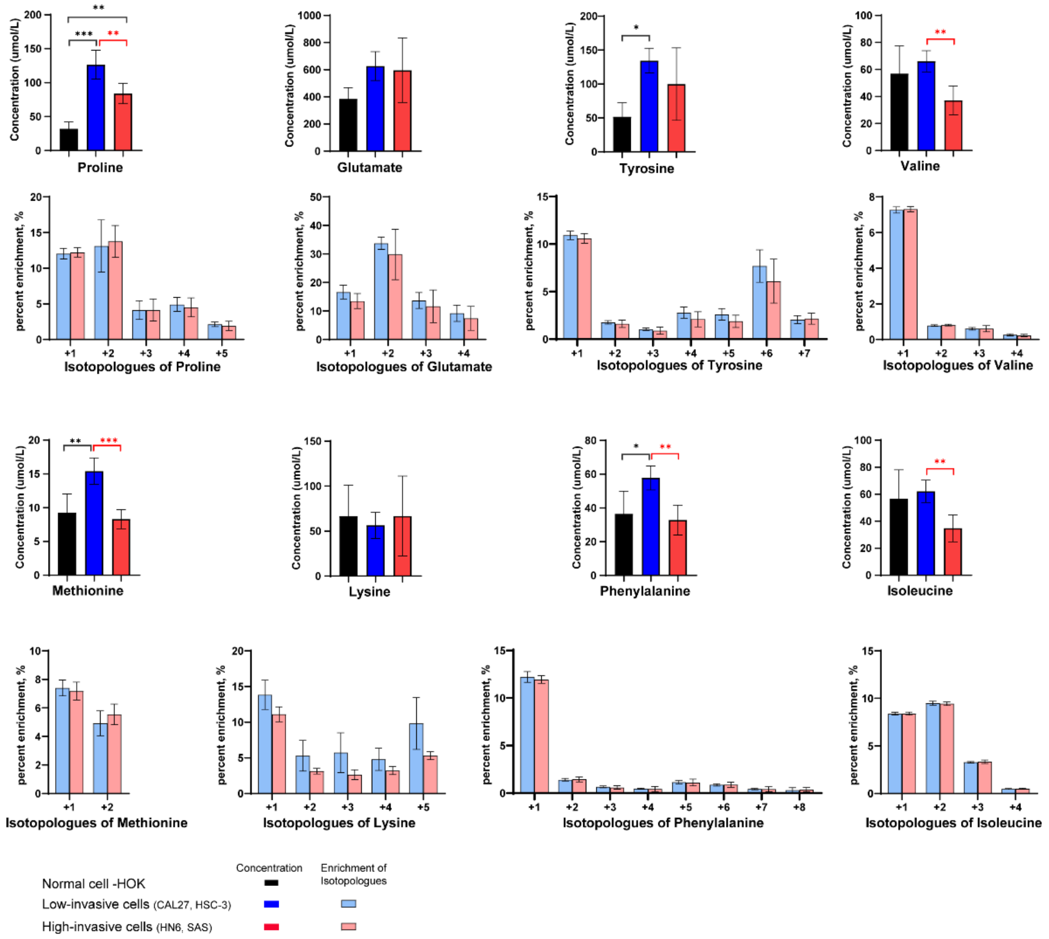

Supplementary Figure S1: The intracellular animo acids metabolic differences between high-invasive cells (HN6, SAS) and low-invasive cells (CAL27, HSC-3). Bar plots demonstrated absolute concentrations of amino acids. Black represents normal cell, blue represents low-invasive cells, red represents high-invasive cells. Pink and light blue bar plots showed the enrichment fractions of ^{13}C-amino acids from [U-$^{13}C_6$] glucose.
